# Cardiotoxicity is mitigated after a supervised exercise program in HER2-positive breast cancer undergoing adjuvant trastuzumab

**DOI:** 10.3389/fcvm.2022.1000846

**Published:** 2022-09-23

**Authors:** Quentin Jacquinot, Nathalie Meneveau, Antoine Falcoz, Malika Bouhaddi, Pauline Roux, Bruno Degano, Marion Chatot, Elsa Curtit, Laura Mansi, Marie-Justine Paillard, Fernando Bazan, Loïc Chaigneau, Erion Dobi, Guillaume Meynard, Dewi Vernerey, Xavier Pivot, Fabienne Mougin

**Affiliations:** ^1^Regional Federative Cancer Institute of Franche-Comté, Besançon, France; ^2^Research Unit EA3920, University of Franche-Comté, Besançon, France; ^3^Department of Medical Oncology, University Hospital, Besançon, France; ^4^UMR 1098, Methodology and Quality of Life Unit in Oncology, University Hospital, Besançon, France; ^5^Physiology-Functional Explorations, University Hospital, Besançon, France; ^6^Heart-Lung Unit, Department of Physiology-Functional Explorations, University Hospital, Grenoble, France; ^7^Department of Cardiology, University Hospital, Besançon, France; ^8^INSERM UMR 1098, Host-Graft-Tumor Interaction, Cell and Gene Engineering, University of Franche-Comté, Besançon, France; ^9^Strasbourg Cancer Institute, Strasbourg, France; ^10^Sports Science Faculty, University of Franche-Comté, Besançon, France

**Keywords:** breast cancer, HER2 overexpression, cardiotoxicity, supervised exercise program, prevention, supportive care

## Abstract

**Background:**

Trastuzumab is used, alone or in conjunction with standard chemotherapy, to treat HER2-positive breast cancer (BC). Although it improves cancer outcomes, trastuzumab. can lead to cardiotoxicity. Physical exercise is a safe and effective supportive therapy in the management of side effects, but the cardioprotective effects of exercise are still unclear.

**Objectives:**

The primary aim of this study was to test whether trastuzumab-induced cardiotoxicity [left ventricular ejection fraction (LVEF) under 50%, or an absolute drop in LVEF of 10%] was reduced after a supervised exercise program of 3 months in patients with HER2-positive breast cancer. Secondary endpoints were to evaluate (i) cardiotoxicity rates using other criteria, (ii) cardiac parameters, (iii) cardiorespiratory fitness and (iv) whether a change in LVEF influences the cardiorespiratory fitness.

**Methods:**

89 women were randomized to receive adjuvant trastuzumab in combination with a training program (training group: TG; *n* = 46) or trastuzumab alone (control group: CG; *n* = 43). The primary and secondary endpoints were evaluated at the end of the supervised exercise program of 3 months (T3).

**Results:**

After exercise program, 90.5 % of TG patients and 81.8% of CG patients did not exhibit cardiotoxicity. Furthermore, whatever the used criterion, percentage of patients without cardiotoxicity were greater in TG (97.6 and 100% respectively) than in CG (90.9 and 93.9% respectively). LVEF and GLS values remained stable in both groups without any difference between the groups. In contrast, at T3, peak VO_2_ (+2.6 mL.min^−1^.kg^−1^; 95%CI, 1.8 to 3.4) and maximal power (+21.3 W; 95%CI, 17.3 to 25.3) increased significantly in TG, whereas they were unchanged in CG (peak VO_2_: +0.2 mL.min^−1^.kg^−1^; 95%CI, −0.5 to 0.9 and maximal power: +0.7 W, 95%CI, −3.6 to 5.1) compared to values measured at T0. No correlation between LVEF changes and peak VO_2_ or maximal power was observed.

**Conclusion:**

A 12-week supervised exercise regimen was safe and improved the cardiopulmonary fitness in particular peak VO_2_, in HER2-positive BC patients treated with adjuvant trastuzumab therapy. The study is under powered to come to any conclusion regarding the effect on cardiotoxicity.

**Clinical trial registration:**

www.ClinicalTrials.gov, identifier: NCT02433067.

## Introduction

Breast cancer (BC) is the most frequently diagnosed cancer and the leading cause of cancer death among women (1). The human epidermal growth receptor-2 (HER2) is overexpressed and/or amplified in approximately 20–25% of BC patients (2). Trastuzumab, a humanized monoclonal antibody against the extracellular domain of HER2, improves disease-free and overall survival in patients with BC overexpressing HER2 (3–6). Although well tolerated, trastuzumab may have adverse cardiac effects, ranging from an asymptomatic drop in left ventricular ejection fraction (LVEF) to symptoms of heart failure (7–9).

There is no universal consensus on the definition of cardiotoxicity. In clinical trials, and according to several professional societies, different thresholds of change in LVEF are used to define cardiotoxicity (2, 10–13). Cardiac dysfunction is considered either as (i) LVEF below 50% or an absolute decrease of 10% from baseline, or (ii) LVEF below 50%, or (iii) an absolute decrease in LVEF of more than 15% from baseline, with LVEF remaining above 50% (11), or (iv) 10% subclinical and asymptomatic reduction in LVEF from baseline (2). Thus, baseline evaluation of cardiac function is recommended prior to initiation of trastuzumab-based therapy and regularly during trastuzumab treatment.

Currently, to assess LVEF before, during and after chemotherapy or trastuzumab treatment, echocardiography is the main non-invasive method for detecting myocardial dysfunction (14). The main advantages of this method are its wide availability, lack of radiation, and its ability to assess hemodynamics and other cardiac structures. Furthermore, echocardiography makes it possible to assess not only 3D-based LVEF, but also left ventricular (LV) global longitudinal strain (GLS). Indeed, GLS was proposed as an early marker of imminent cardiotoxicity (14, 15). A reduction of 15% during chemotherapy is associated with a higher probability of significant left ventricular systolic dysfunction (14, 15).

Adjuvant therapies for BC may induce a cascade of cardiac dysfunction, starting with LV alterations, resulting in LVEF decrease and abnormal LV contractility, a stroke volume reduction and cardiac output, and ultimately, a decrease in nutrient and oxygen supply (16, 17). These alterations may lead to dyspnea, lower gas diffusion capacity, thereby compromising the oxygen supply and elimination of carbon dioxide (18, 19). In addition, cardiotoxicity is accompanied by a decrease in cardiorespiratory capacities, which in turn exacerbate exercise intolerance and deconditioning (20–22). It is therefore essential to prevent cardiotoxicity as early as possible, from the initiation through to the end of treatment, and beyond (23–25).

Physical exercise is increasingly recognized as an effective non-pharmacological approach to counteracting the adverse effects of cancer therapy (26–35). Nevertheless, data are sparse regarding the effects of physical exercise on cardiac toxicity induced by adjuvant treatment with trastuzumab. Besides, Murray et al., reported recently that the role of exercise on cancer treatment-related cardiac dysfunction is still unclear (36).

To date, considering the small number of available data and the heterogeneity between studies, there is inconclusive evidence of a cardioprotective effect of physical activity in patients receiving adjuvant trastuzumab. Therefore, the primary aim of this study was to ascertain whether a supervised exercise training program lasting 3 months would reduce trastuzumab-induced cardiotoxicity (TIC) in HER2-positive breast cancer undergoing adjuvant trastuzumab. Secondary objectives were to assess (i) cardiotoxicity defined using various different criteria; (ii) cardiac parameters (LVEF and GLS); and (iii) cardiorespiratory fitness (peak VO_2_ and maximal power). We hypothesized that a supervised exercise program would be effective in preventing TIC and improving cardiorespiratory fitness.

## Materials and methods

### Ethics approval

The CARDAPAC study was conducted in compliance with the Declaration of Helsinki. It received approval by the Ethics Committee (Comité de Protection des Personnes Est-II), Besançon, France under the number P/2014/241 and by the National Health Products Safety Agency (N° ID RCB 2014-A01911-46). The trial was registered on ClinicalTrials.gov under the number NCT02433067. Financial support was provided by the Ligue Contre le Cancer association (CCIR-GE).

### Study design

We performed the present phase II, randomized, prospective, multicentre, non-comparative trial, and included patients from 5 sites in Eastern France (one university teaching hospital, three non-academic public hospitals and one private clinic). Detailed methods have been published elsewhere (37).

Briefly, women were recruited based on the eligibility criteria summarized in Table 1. To be included, patients had to be aged 18 to 85 years, had a first HER2-positive breast cancer, histologically confirmed with a WHO performance status ≤ 1, had to have completed chemo-radiotherapy, had to have a normal cardiac function with LVEF ≥ 50% (less than 3 months) and had to present a certificate of non-contraindication to the practice of physical activity. All participants provided written informed consent prior to enrolment. Patients were randomly assigned in a 1:1 ratio to receive adjuvant trastuzumab in combination with a supervised training exercise program (training group, TG) or trastuzumab alone without exercise program (control group, CG). Randomization was performed according to the minimization technique with stratification (eRandomisation software Tenalea^®^) by age (18–30 vs. 30–50 vs. 50–85 years) and global health score defined from a quality of life questionnaire [QLQ-C30 (0–30 vs. 30–50 vs. 50–70 vs. >70)].

**Table 1:** Inclusion and exclusion criteria.

**Inclusion criteria**	**Exclusion criteria**
- Patients aged 18 to 85 years - First HER2 positive breast cancer, confirmed histologically - WHO Performance status ≤ 1 - Completed chemo-radiotherapy - Normal renal function (creatinine clearance ≥ 60 ml.min^−1^) - Normal heart function with LVEF ≥ 50% (As assessed by echocardiography dating from less than 3 months previously) - Normal liver function (normal ASAT and ALAT) - Certificate of non-contraindication to the practice of physical activity - Active contraception or menopaused	- HER2 negative breast cancer - Patients with metastases - Heart failure (LVEF ≤ 50%) - Resting oxygen saturation (SaO_2_) ≤ 92% - Autoimmune disease (systemic lupus erythematosus, rheumatoid arthritis) - Symptomatic osteoarthritis, cardiovascular disease (angina or uncontrolled hypertension) or lung disease (chronic obstructive pulmonary disease) - Patients suffering from malnutrition (BMI < 18 kg.m^−2^) or weight loss of >10% during the previous 3 months - Patients with psychiatric or cognitive disorders deemed unsuitable for physical activity - Pregnant or breastfeeding patients

ALAT, alanine aminotransferase; ASAT, aspartate transaminase; BMI, body mass index; HER2, human epidermal growth factor receptor 2; LVEF, left ventricular ejection fraction; SaO_2_, oxygen arterial saturation; WHO, world health organization.

### Study protocol

All evaluations were carried out at enrolment (T0), and at three (T3) and 6 months (T6). Between T0 and T3, both groups received standard oncological care either with (TG) or without (CG) supervised exercise program. Between T3 and T6, both groups had standard oncological care without supervised physical activity.

#### Cardiorespiratory exercise testing

Cardiorespiratory exercise testing was conducted using a cyclo-ergometer (Ergoselect 200; Ergoline; Bitz, Germany) under the supervision of a respiratory medicine specialist. The assessor provided standardized encouragement until maximal power was reached. After a 3-min warm-up period at a power of 30 watts, intensity was gradually increased by 10 watts every min until the patient was exhausted or limited by factors such as fatigue, refusal to continue the exercise, and/or appearance of cardiac symptoms (i.e., ECG abnormalities or arterial hypertension). The cadence was maintained between 50 and 70 revolutions per min until the tolerated power. The highest oxygen achieved during the final 60 seconds of the test was considered as peak (peak VO_2_). Active recovery was pursued for 10 min, at the same power as that employed during the warm-up.

Heart rate (HR) was continuously monitored, from rest to the end of recovery, with a 12-lead electrocardiogram (CASE P2, GE Healthcare, Buckinghamshire, UK).

The ventilatory parameters [ventilation per (VE), respiratory rate (RT), tidal volume (VT)], oxygen consumption (VO_2_) and carbon dioxide release (VCO_2_)] were recorded continuously, using a gas exchange analyzer system (MGC-CPX System; MGC Diagnostics Corporation, Saint Paul, MN, USA), which was calibrated using gases of known concentration. Ventilatory variables were averaged every 30 seconds.

The first and second ventilatory thresholds (VT_1_ and VT_2_) were assessed from the relation between the respiratory exchange ratios (VCO_2_/VO_2_, VE/ VO_2_, VE/ VCO_2_) by two experts, in a blinded fashion, using the V-slope method according the Wasserman method (38). The mean of the two closest values was taken as the VT and the corresponding power (watts) was recorded. Power corresponding at VT_1_ and VT_2_ was used to guide intensity for the supervised exercise program.

#### Supervised exercise program

Patients allocated to the TG performed a supervised exercise training, which was carried out on a cycloergometer, with electromagnetic braking, and comprised three sessions of 55 min per week for 12 weeks, giving a total of 36 sessions. Each session began with 5 min of warm-up at an intensity equal to half the power of VT_1_ ($\frac{1}{2}$ VT_1_), followed by 9 work bouts of 5 min each, for a maximum total exercise time of 45 min. Each 5-min work bout consisted of 4 min of moderate intensity power, denoted “base”, followed by a 1-min-high intensity work bout denoted “peak”. Initially, the “base” exercise level was chosen as the VT_1_ power and the “peak” level as the VT_2_ power. At the end of the last “peak”, an active recovery period of 5 min was performed at the same power as that of the warm-up ($\frac{1}{2}$ VT_1_).

The exercise program was supervised by an adapted physical activity specialist to ensure the safety and quality of the program. During each session, HR was continuously measured with a fingertip pulse oximeter (Onyx^®^ Vantage 9590, Nonin Medical, Inc., USA), at the end of the warm-up, at the end of each “base” and each “peak”, as well as every min during active recovery. The peak and base loads were alternately readjusted by 10 watts when values of HR at the end of the session were 10 to 12 beats/min below the target heart rate.

It should be noted that the proposed exercise intervention was never intended to replace or interfere with the standard of care.

### Primary endpoint

**Cardiotoxicity** was defined as either a decrease of the LVEF under 50% at T3 (independently of the baseline value) or an absolute drop in LVEF of at least 10% from T0 to T3 (criterion 1).

All echocardiographic measurements were performed in the supine position, on 3 representative beats, and data were averaged. Echocardiographic acquisitions were performed by a single experienced cardiologist blinded to the patient assignment group, to avoid measurement bias.

LVEF was assessed by transthoracic Doppler echocardiography (Philips EPIQ7, Philips Healthcare, Andover, MA, USA) in the apical 4-chamber (4C) view using Simpson's biplane rule, according to the American Society of Echocardiography recommendations (35, 36).

### Secondary endpoints

#### Cardiotoxicity defined using other criteria

Due to the different criteria used in numerous clinical trials (2, 10–13), cardiotoxicity was also assessed using other criteria, namely: (i) LVEF less than or equal to 50% at T3 or an absolute decrease in LVEF of at least 15% from T0 to T3 (criterion 2) and (ii) LVEF less than 50% at T3 (criterion 3).

Additionally, an absolute decrease in GLS of 15% from T0 to T3 was used as the 4^th^ criterion for cardiotoxicity (39–41). GLS was assessed in the basal, mid-ventricular and apical segments in the 4-chamber view. Since the recommendations of the European Society of Cardiology were only published in 2016 (42), GLS was measured only from the second year of inclusion onwards.

#### Other outcomes

Cardiac parameters were evaluated by studying baseline LVEF and GLS and cardiopulmonary functions were assessed by maximal power (watts), peak VO_2_ (ml.min^−1^.kg^−1^), heart rate (beats.min^−1^) and VO_2_/HR (mL. beats^−1^) as described above.

Furthermore, two subgroups were set-up: TOX and NoTOX according to the presence or absence of cardiotoxicity as defined by criterion 1.

### Sample size

Since the beginning of the study and the publication of the design (37), the sample size was modified, in agreement with methodologists and authors for two reasons: (i) a low rate of inclusion (1.5 patients/month) despite 5 years of inclusion, (ii) the recent introduction of targeted therapies, in particular trastuzumab emtansine (T-DM1), an antibody drug combining the anticancer properties of trastuzumab and the antineoplastic cytotoxic drug DM1 (43–45). Patients receiving this treatment could not be included, reducing the active supply of patients eligible for our study. The power initially was set at 90% but was decreased to 80% to guarantee the feasibility of the research.

In the experimental arm, according to Fleming's one-stage design with a one-sided alpha risk of 5% and power of 80%, 45 patients were required in the TG to test the following hypotheses:

- H0 (Null): A cardiotoxicity free rate at 3 months of 75% (uninteresting).
- H1 (Alternative): A cardiotoxicity free rate at 3 months of 90% (warranting further investigation in phase III trial).

These hypotheses were based on cardiotoxicity rates observed in randomized clinical trials, which were approximately 13 to 27%, depending on the molecules used during chemotherapy (10).

The decision rule involves 45 evaluable patients in the TG (a patient was considered evaluable when LVEF data was available at T0 and T3), with a 3-month follow-up from randomization:

- If 38 or fewer patients were free of cardiotoxicity at 3 months (84.4%), the supervised exercise program was declared to be of limited interest,
- If 39 or more patients were free of cardiotoxicity at 3 months (86.7%), the supervised exercise program was declared to warrant further phase III evaluation.

CG group served as calibration to validate the H0 hypothesis.

### Statistical analysis

Qualitative variables were described as number and percentage, and quantitative variables as median with interquartile range (IQR) or median with range (Min-Max) for age. Echocardiography variables and cardiorespiratory parameters at maximal exercise were described as mean ± standard deviation (SD) at T0, T3 and T6. Differences over time were described as the difference in means, with 95% confidence interval (CI). Violin plots were used to obtain a longitudinal representation of data during follow up. The Wilcoxon-Mann-Whitney test was used to compare median values of LVEF (according to criterion 1) and cardiorespiratory parameters of patients according to the presence or absence of cardiotoxicity at T0 and T3. Spearman's correlation coefficient was used to assess the association between changes in LVEF, peak VO_2_ and maximal power.

Statistical analysis was performed using SAS version 9.4 (SAS Institute Inc., Cary, NC, USA) and R software version 4.0.3 (R Development Core Team, Vienna, Austria; http://www.r-project.org).

## Results

### Population

At enrolment, all patients had completed chemotherapy and radiotherapy. They were treated only with trastuzumab in adjuvant for 12 months with a total of 18 cycles, as already described in detail in Jacquinot et al. (37).

**Figure 1:**
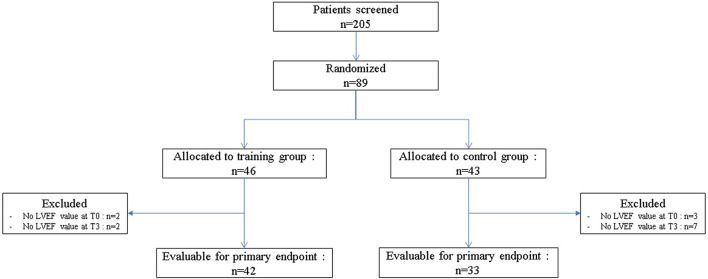
The study flow chart.

Between April 2015 and February 2020, among of 205 patients screened, 89 were randomized, 46 patients (51.7%) to the TG and 43 patients (48.3%) to the CG (Figure 1). The baseline clinical characteristics of all participants are presented in Table 2. The median age was 51.0 years (IQR: 43.4–55.8) and median body mass index was 25 kg.m^−2^ (IQR: 22.5–28.9). The average score obtained from the QLQ-C30 questionnaire, which evaluates various dimensions of health-related quality of life and global health, was 66.7/100 (IQR: 54.2–75.0).

**Table 2:** Patient's characteristics at inclusion.

	**Overall population**		**Training group**		**Control group**	
	***n* = 89**		***n* = 46**		***n* = 43**	
	**n**	**%**	**n**	**%**	**n**	**%**
**STRATIFICATION CRITERIA**						
**Age (years)**	
Median (IQR)	51.0 (43.4–55.8)		51.1 (43.1–55.8)		51.0 (43.4–56.4)	
Median [range]	51.0 [28.7–74.5]		51.1 [28.7–66.8]		51.0 [32.6–74.5]	
**Age (years)**			
18–30	1	1.1	1	2.2	0	0
30–50	40	44.9	21	45.7	19	44.2
50–85	48	53.9	24	52.2	24	55.8
**Global health score** - **QLQ-C30**						
Median [range]	66.7 [25.0–100.0]		66.7 [25.0–100.0]		66.7 [41.7–100.0]	
Missing	9		4		5	
**Global health score** - **QLQ-C30**			
0–30	2	2.5	2	4.8	0	0
31–50	18	22.5	10	23.8	8	21.1
51–70	31	38.8	16	38.1	15	39.5
71–100	29	36.2	14	33.3	15	39.5
Missing	9	-	4	-	5	-
**DISEASE AND TREATMENT CHARACTERISTICS**						
**Scarff-Bloom-Richardson grade**						
I	4	4.6	3	6.7	1	2.4
II	32	36.8	17	37.8	15	35.7
III	51	58.6	25	55.6	26	61.9
Missing	2	-	1	-	1	-
**ER**						
+	58	65.2	33	71.7	25	58.1
–	31	34.8	13	28.3	18	41.9
**PR**						
+	41	46.1	24	52.2	17	39.5
–	48	53.9	22	47.8	26	60.5
**HER2**						
+	89	100	46	100	43	100
**Type of surgery**						
Mastectomy	25	28.1	13	28.3	12	27.9
Conservative surgery	64	71.9	33	71.7	31	72.1
**Chemotherapy cycles**						
Anthracyclines + taxanes	71	79.8	36	78.3	35	81.4
Anthracyclines without taxanes	1	1.1	0	-	1	2.3
Without anthracyclines	17	19.1	10	21.7	7	16.3
**Radiotherapy**			
Yes	78	88.6	39	86.7	39	90.7
No	10	11.4	6	13.3	4	9.3
Missing	1	-	1	-	0	-
**Trastuzumab cycles**						
18	89	100	46	100	43	100
**LIFE HABITS**						
**Tobacco consumption**						
Smoker	16	18.0	11	23.9	5	11.6
Non smoker	57	64.0	33	71.7	24	55.8
Ex-smoker	16	18.0	2	4.4	14	32.6
**Alcohol consumption**						
Yes	43	48.9	22	48.9	21	48.9
No	45	51.1	23	51.1	22	51.1
Missing	1	-	1	-	0	-
**Analgesic medication**						
Yes	23	25.8	13	28.3	10	23.3
No	66	74.2	33	71.7	33	76.7
**Physical activity**						
Yes	84	94.4	43	93.5	41	95.4
No	5	5.6	3	6.5	2	4.6
**Physical activity level**						
Less than 1h/week/year	33	39.8	12	28.6	21	51.2
1h to 3h/week/year	39	47.0	21	50.0	18	43.9
More than 3h/week/year	11	13.3	9	21.4	2	4.9
Missing	1	-	1	-	0	-
**ANTHROPOMETRIC VARIABLES**						
**Height (cm)**						
Median (IQR)	163 (159–169)		163.0 (159.0–166.0)		166.0 (160.0–173.0)	
Missing	1		1		0	
**Body mass (kg)**						
Median (IQR)	67.5 (60.0–78.0)		65.2 (60.0–71.0)		72.2 (60.0–84.3)	
**Body Mass Index (kg.m** ^**−2**^ **)**						
Median (IQR)	25.0 (22.5–28.9)		24.7 (22.3–28.4)		26.0 (22.7–30.1)	
Missing	1		1		0	

ER, Estrogen Receptor; HER2, human epidermal growth factor receptor 2; PR, Progesterone Receptor; QLQ-C30, Quality of Life Questionnaire – Core 30.

Among the overall population of 89 patients, 58 (65.2%) were estrogen positive, 64 (71.9%) had a conservative surgery and 78 (88.6%) had received radiotherapy. Chemotherapy regimens were similarly distributed between the two groups. Indeed, 71 patients (79.8%) were treated sequentially with chemotherapy based on anthracyclines and taxanes, 17 patients (19.1%) with anthracyclines without taxanes and one patient (1.1%) without anthracyclines. All patients also received trastuzumab concomitantly to taxanes. Among the 89 patients, 57 (64%) were non-smokers, 45 (51.1%) did not consume alcohol and 66 (74.2%) did not take analgesic medication. Furthermore, 84 patients (94.4%) regularly performed physical activity, 33 of them (39.8%) practiced less than 1 h a week, 39 (47%) at least 1 to 3 h a week and 11 (13.3%) more than 3 h per week.

### Cardiotoxicity

For the primary endpoint, 42 (91.3%) patients were evaluable in TG (4 patients excluded) and 33 (76.3%) in CG (10 patients excluded; Figure 1). In TG, although we did not reach the target of 45 evaluable patients randomized, to observe at least 39 patients free of cardiotoxicity, we nevertheless observed 38 patients free of cardiotoxicity at T3, among the 42 evaluable patients randomized [90.5%, 90%CI (79.5–96.7)]. Patients, who presented a cardiotoxicity, had an absolute decrease of LVEF at least 10% but none of them had a LVEF < 50% (Table 3). The lower limit of the binomial 90% confidence interval for the rate of patients free of cardiotoxicity at T3 in TG is higher than the H0 hypothesis rate (75%) demonstrating that this estimation is clearly above 75% and that the intervention can therefore be considered to warrant further evaluation.

**Table 3:** Study of cardiotoxicity at T3 according to the different criteria, in patients of both groups (TG and CG).

	**Overall population**		**Training group**		**Control group**	
	***n* = 75**		***n* = 42**		***n* = 33**	
	**n**	**%(90%CI)**	**n**	**%(90%CI)**	**n**	**%(90%CI)**
**Cardiotoxicity (Criterion 1)**			
Yes	10	13.3 (7.4–21.6)	4	9.5 (3.3–20.5)	6	18.2 (8.2–32.8)
No	65	86.7 (78.4–92.6)	38	90.5 (79.5–96.7)	27	81.8 (67.2–91.8)
**If yes, reason ?**						
Absolute decrease of at least 10%	8	80.0	4	100	4	66.7
LVEF at T3 < 50%	2	20.0	0	0.0	2	33.3
**Cardiotoxicity (Criterion 2)**						
Yes	4	5.3 (1.8–11.8)	1	2.4 (0.0–10.8)	3	9.1 (2.5–21.8)
No	71	94.7 (88.2–98.2)	41	97.6 (89.2–100)	30	90.9 (78.2–97.5)
**If yes, reason?**						
Absolute decrease of at least 15%	2	50.0	1	100	1	33.3
LVEF at T3 < 50%	2	50.0	0	0.0	2	66.7
**Cardiotoxicity (Criterion 3)**						
Yes	2	2.7 (0.5–8.2)	0	0.0 (0.0–7.0)	2	6.1 (1.1–17.9)
No	73	97.3 (91.8–99.5)	42	100 (93.0–100)	31	93.9 (82.1–98.9)
**Cardiotoxicity (Criterion 4)**						
Yes	10	22.7	4	16.7	6	30.0
No	34	77.3	20	83.3	14	70.0
Missing	31	-	18	-	13	-

LVEF, left ventricular ejection fraction.

In the control group, at T3, 27 patients were free of cardiotoxicity among the 33 evaluable patients randomized [81.8%, 90%CI (67.2–91.8)]. Among of 6 patients who presented a cardiotoxicity (18.2%), an absolute decrease of LVEF at least 10% in 4 patients and a LVEF < 50% in 2 patients were observed. The non-comparative context of the study did not provide sufficient statistical power to demonstrate a significant difference in the rate of cardiotoxicity at T3 between the two groups.

Regarding the secondary endpoints, based on criterion 2, 97.6% (*n* = 41) in TG and 90.9% (*n* = 30) in CG did not experience cardiotoxicity. Indeed, according to this criterion, one patient from each group presented an absolute decrease of LVEF by at least 15% and 2 patients in CG a LVEF <50%. Furthermore, using criterion 3, 100% (*n* = 42) of patients in TG and 93.9% (*n* = 31) in CG were free of cardiotoxicity (Table 3), only 2 patients from CG having presented a LVEF <50%. Whatever the criterion (1, 2 or 3), there were consistently more patients without cardiotoxicity in TG compared to those in CG. According to criterion 4 (absolute drop in GLS of 15% from baseline), 83.3% (*n* = 20) of patients in TG did not have cardiotoxicity vs. 70 % (*n* = 14) in CG (Table 3).

### Cardiac parameters

At inclusion (T0), median values of LVEF [TG: 61.6 % (6.9); CG: 59.6 % (6.7)] and GLS [TG: −20.8 % (2.6); CG: −19.3 (2.0)] were within normal range, greater than or equal to 50 or −18% respectively (Table 4).

**Table 4:** Evaluation of cardiac parameters.

	**Training group (*****n*** = **42)**										**Control group (*****n*** = **33)**									
	**T0**		**T3**		**T6**		**T3-T0**		**T6-T3**		**T0**		**T3**		**T6**		**T3-T0**		**T6-T3**	
	***n***	**Mean (SD)**	***n***	**Mean (SD)**	***n***	**Mean (SD)**	***n***	**Mean (95% CI)**	***n***	**Mean (95% CI)**	***n***	**Mean (SD)**	***n***	**Mean (SD)**	***n***	**Mean (SD)**	***n***	**Mean (95% CI)**	***n***	**Mean (95% CI)**
LVEF (%)	42	61.6 (6.9)	42	61.7 (6.5)	30	61.5 (6.3)	42	0.1 (−2.6, 2.8)	30	−0.9 (−4.3, 2.6)	33	59.6 (6.7)	33	59.0 (5.5)	31	61.2 (6.9)	33	−0.7 (−3.0, 1.6)	31	2.1 (−0.3, 4.5)
GLS (%)	24	−20.8 (2.6)	29	−20.1 (1.8)	25	−20.2 (1.9)	24	0.5 (−0.8, 1.8)	20	0.1 (−0.9, 0.8)	21	−19.3 (2.0)	21	−21.3 (2.9)	26	−19.8 (2.6)	20	−1.6 (−2.7, −0.4)	20	0.7 (−0.8, 2.2)

LVEF, left ventricular ejection fraction; GLS, left ventricular global longitudinal strain.

**Figure 2:**
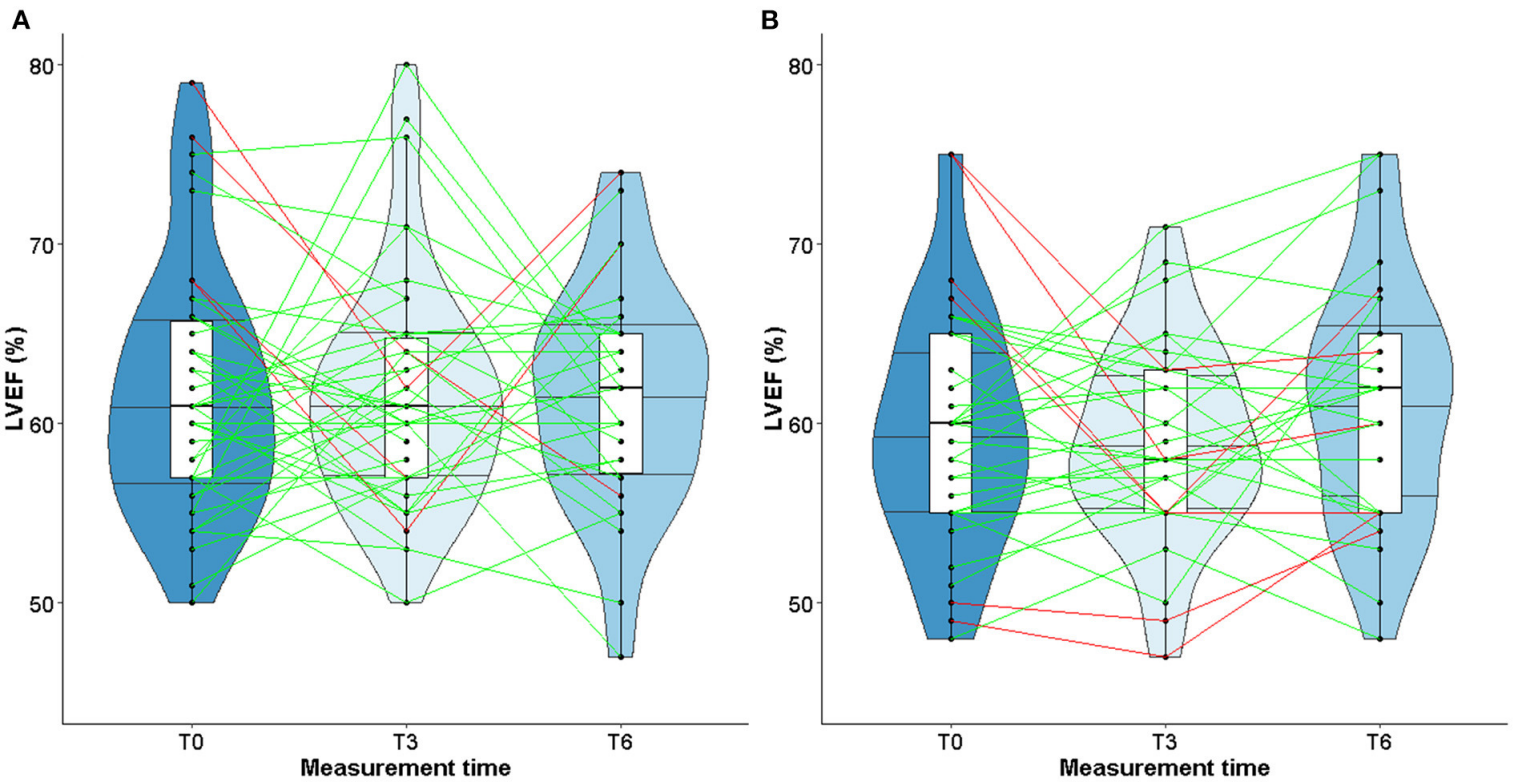
Violin plot for evolution of LVEF in TG **(A)** and CG **(B)**.

At T3 and T6, values remained stable both in TG or CG, with similar medians (Table 4). The violin plots in Figure 2 show the changes in LVEF between T0, T3, and T6 with heterogeneous individual trajectories in both groups.

### Cardiorespiratory variables

The training program was well tolerated overall, and that no adverse effects were observed in the patients in TG.

At T3, peak VO_2_ increased significantly in TG (mean difference, 2.6 mL.min^−1^.kg^−1^; 95% CI, 1.8 to 3.4) whereas it was unchanged in CG (mean difference, 0.2 mL.min^−1^.kg^−1^; 95% CI, −0.5 to 0.9; Table 5). Similarly, maximal power increased in TG (mean difference 21.3 W; 95% CI, 17.3 to 25.3), whereas it did not change in CG (mean difference 0.7 W; 95% CI,−3.6 to 5.1; Table 5). VO_2_/HR ratio increased significantly in TG (mean difference 0.7 mL.beats^−1^; 95% CI, 0.3, 1.1) whereas it remained unchanged in CG (mean difference 0.1 mL.beats^−1^; 95% CI, −0.2, 0.4). Furthermore, in both groups, HR reached the maximal predicted value and failed to show any difference (TG: mean difference 2.5 beats.min^−1^; 95% CI, −2.8 to 7.7 and CG: mean difference −1.4 beats.min^−1^; 95% CI, −5.7 to 3.0).

**Table 5:** Cardiorespiratory variables at maximal exercise.

	**Training group (*n* = 42)**										**Control group (*n* = 33)**									
	**T0**		**T3**		**T6**		**T3-T0**		**T6-T3**		**T0**		**T3**		**T6**		**T3-T0**		**T6-T3**	
	***n***	**Mean (SD)**	***n***	**Mean (SD)**	***n***	**Mean (SD)**	***n***	**Mean (95% CI)**	***n***	**Mean (95% CI)**	***n***	**Mean (SD)**	***n***	**Mean (SD)**	***n***	**Mean (SD)**	***n***	**Mean (95% CI)**	***n***	**Mean (95% CI)**
Peak VO_2_ (mL.min^−1^.kg^−1^)	41	25.7 (6.1)	35	28.4 (6.1)	27	26.4 (6.2)	35	2.6 (1.8, 3.4)	26	−1.0 (−1.8, −0.2)	33	24.3 (8.3)	28	24.2 (6.6)	26	23.8 (6.9)	28	0.2 (−0.5, 0.9)	24	0.4 (−0.5, 1.2)
Maximal power (watts)	41	119.2 (23.7)	34	138.7 (27.7)	26	134.7 (32.3)	34	21.3 (17.3, 25.3)	24	−5.0 (−9.3, −0.8)	32	118.4 (25.5)	29	120.1 (30.2)	26	121.8 (27.2)	29	0.7 (−3.6, 5.1)	24	4.1 (−0.2, 8.5)
HR (beats.min^−1^)	40	169.1 (17.8)	35	170.5 (21.5)	27	170.4 (17.5)	34	2.5 (−2.8, 7.7)	26	1.8 (−4.5, 8.1)	33	164.0 (15.0)	29	163.7 (13.6)	26	167.9 (13.8)	29	−1.4 (−5.7, 3.0)	24	5.7 (0.2, 11.2)
VO_2_/HR (mL.beats^−1^)	40	10.1 (1.7)	35	10.8 (1.6)	27	10.5 (1.7)	34	0.7 (0.3, 1.1)	26	−0.4 (−0.7, −0.0)	33	10.3 (2.4)	28	10.5 (2.4)	26	10.1 (2.4)	28	0.1 (−0.2, 0.4)	24	−0.1 (−0.5, 0.2)

Peak VO_2_, Peak oxygen uptake; HR, Heart Rate; VO_2_/HR, Oxygen pulse.

At T6, peak VO_2_, maximal power, and VO_2_/HR values decreased in TG but were still higher than those measured at T0. Conversely, in CG, these variables were unchanged in comparison with baseline values.

**Figure 3:**
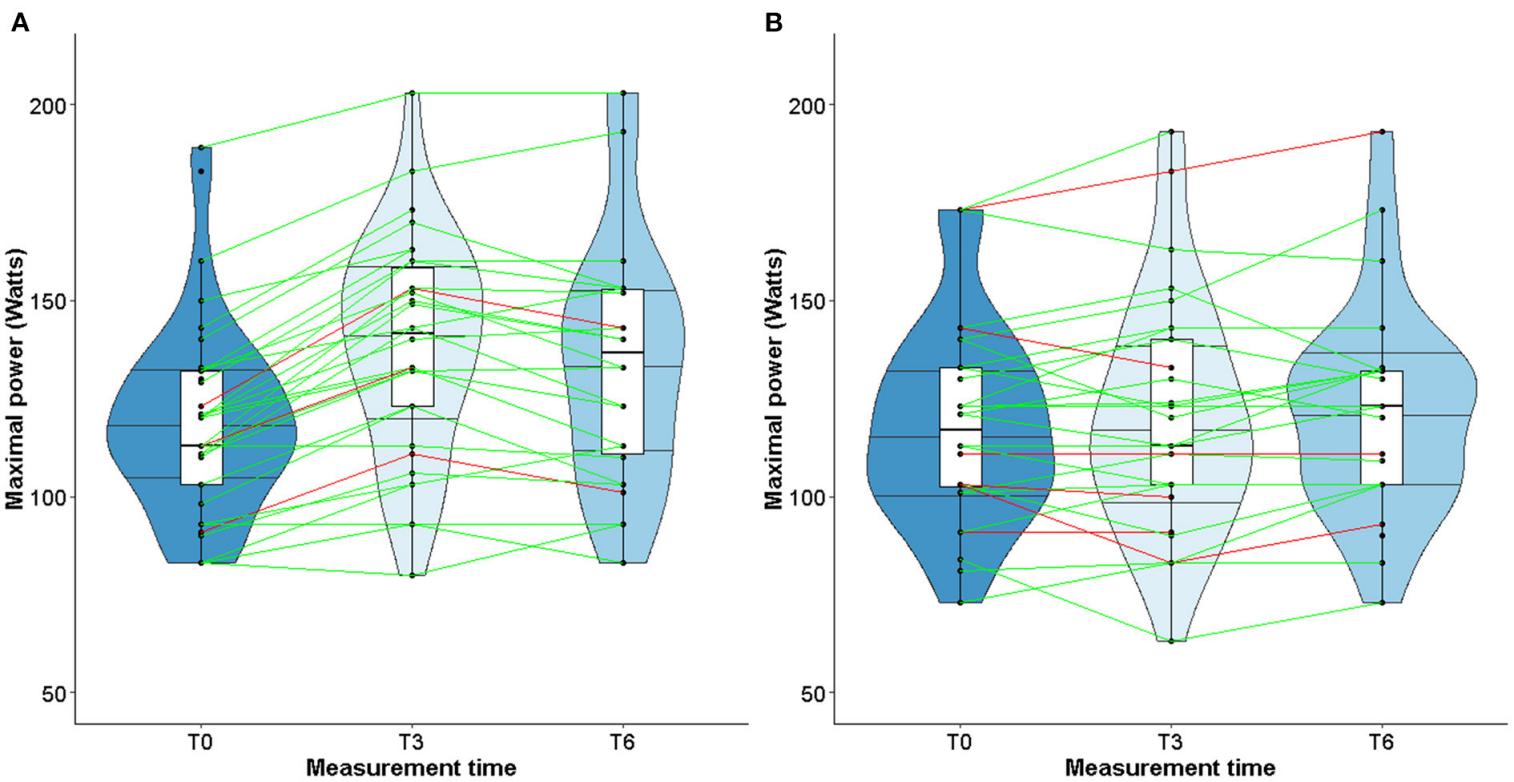
Violin plot for evolution peak VO_2_ in TG **(A)** and CG **(B)**.

**Figure 4:**
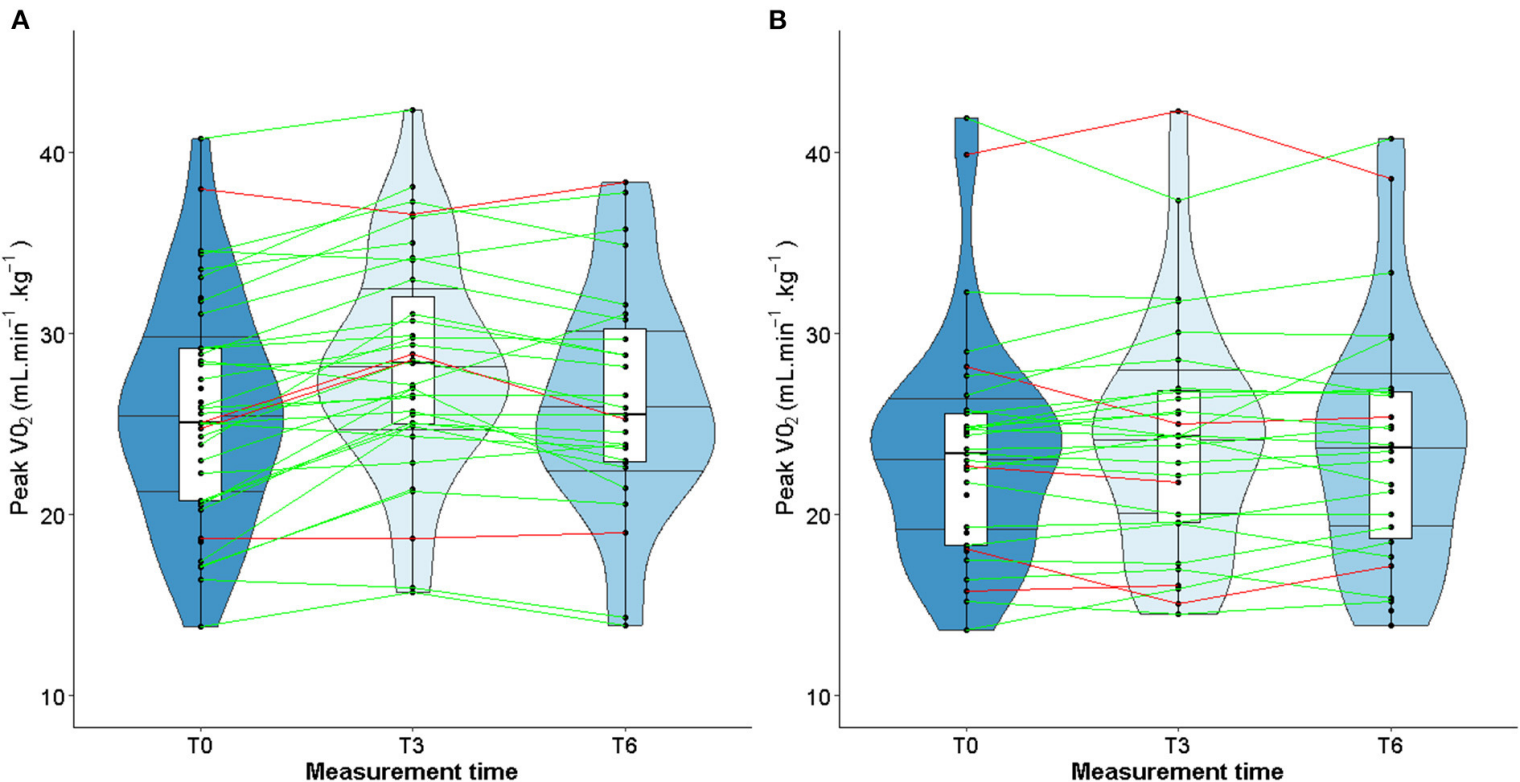
Violin plot for evolution of maximal power in TG **(A)** and CG **(B)**.

When considering only TG patients who presented cardiotoxicity at T3 (shown in red in Figure 3), peak VO_2_ increased in 2 out of 4 patients, remained stable in one patient and declined slightly in one patient (Figure 3A). As for the CG, peak VO_2_ decreased in 3 out of 5 patients and increased in 2 patients (Figure 3B). Unlike LVEF, homogeneous individual trajectories were observed (Figure 3). Furthermore, the same pattern of individual trajectories was observed for the maximal power (Figure 4).

No correlation between LVEF changes and either peak VO_2_ or maximal power was observed (R = 0.035; *p* = 0.78; and R = 0.004; *p* = 0.97 respectively).

### TOX and NoTOX groups

As shown in Table 6, LVEF was significantly higher at T0 in the TOX group [68.0 % (67.0–75.0)] compared to the NoTOX group [60.0% (56.0–63.0); *p* < 0.01]. At T3, it was significantly lower in TOX group [TOX: 56.0 (54.0–62.0)] than in NoTOX group [60.0 (57.0–64.0); (*p* < 0.04)] with a significant relative difference (TOX: −12.0 (−14.0, −11.0) vs NoTOX: 1.0 (−3.0, 5.0); *p* < 0.01).

**Table 6:** Subgroups analysis in patients who presented cardiotoxicity (TOX group) or not (NoTOX group).

	**TOX group** ***n*** **=** **10**			**NoTOX group** ***n*** **=** **65**			***p***		
	**T0**	**T3**	**T3-T0**	**T0**	**T3**	**T3-T0**	**T0**	**T3**	**T3-T0**
**LVEF (%)**									
Median (IQR)	68.0 (67.0–75.0)	56.0 (54.0–62.0)	−12.0 (−14.0,−11.0)	60.0 (56.0–63.0)	60.0 (57.0–64.0)	1.0 (−3.0, 5.0)	< 0.01	0.04	< 0.01
**Peak VO_2_ (mL.min^−1^·kg^−1^)**								
Median (IQR)	23.8 (18.7–28.2)	25.0 (18.7–28.9)	0.0 (−1.4,−2.4)	24.7 (20.6–28.0)	26.5 (22.9–30.1)	1.3 (0.1, 3.8)	0.84	0.64	0.08
Missing	0	1	1	1	11	11			
**Maximal power (watts)**								
Median (IQR)	112.0 (103.0–143.0)	111.0 (100.0–133.0)	0.0 (−3.0, 20.0)	120.0 (103.0–132.0)	132.0 (111.0–153.0)	12.0 (2.0–21.0)	0.95	0.34	0.17
Missing	0	1	1	2	11	11			

LVEF, left ventricular ejection fraction; Peak VO_2_, Peak oxygen uptake.

In contrast, at T0 and T3, no difference was observed between groups with regard to peak VO_2_ and maximal power, although values were numerically slightly lower in the TOX group than in the NoTOX group (Table 6).

## Discussion

Although trastuzumab improves outcomes in patients with HER2-positive breast cancer (BC) (46), trastuzumab-induced cardiotoxicity (TIC) compromises the health-related quality of life and overall survival of cancer patients (47, 48). A progressive LVEF decline and potentially overt heart failure may occur. However, recent findings demonstrated that this form of cardiomyopathy is mostly reversible with early detection and prompt introduction of an appropriate therapeutic strategy (49, 50).

To our knowledge, few studies have investigated the effects of an exercise training program on TIC in BC patients treated by trastuzumab. The CARDAPAC study is one of the first randomized, clinical studies to offer a supervised exercise training with HER2-positive BC patients under adjuvant trastuzumab, to prevent TIC.

The major findings are that an exercise program, combining high and moderate intensities, minimized TIC. Indeed, the rate of patients free of cardiotoxicity observed after a supervised exercise training of 3 months (T3) was 90.5% [90%CI (79.5–96.7)] vs. 81.8% [90%CI (67.2–91.8)] in TG and CG patients respectively. The lower limit of the binomial 90% confidence interval for the rate of patients free of cardiotoxicity at T3 in the TG was higher than the H0 hypothesis rate (75%), demonstrating that this estimation is above 75% and therefore, that the results must be considered worthy of further evaluation. Regardless of the criterion used to defined cardiotoxicity (1, 2, 3 or 4), the number of patients without cardiotoxicity was higher in the TG than in the CG, although the non-comparative nature of the study did not allow sufficient statistical power to demonstrate a significant difference in the rate of cardiotoxicity at T3 between the two groups.

In the literature, Foulkes et al. did not identify cardiotoxicity in trained patients during anthracycline chemotherapy, whereas 25% of non-trained patients were independently diagnosed with cardiotoxicity (51). Their study took place during an anthracycline-based chemotherapy regimen, and only 7 out of 17 patients received treatment with trastuzumab whereas in our study, all patients were treated by adjuvant trastuzumab only at enrolment. In our study, the rate of cardiotoxicity as defined by criterion 1, reached 13.3% in the whole population (9.5% in TG and 18.2% CG). This percentage is consistent with the results of Naumann et al. (52) and Lemieux et al. (53) who observed cardiotoxicity rates of respectively 15.7 and 13.5% in women treated with trastuzumab for primary BC. Those percentages were higher than those reported by Smith et al. (4) and Pivot et al. (11) in BC patients having received also only trastuzumab (5.7 and 3.4% respectively).

These discrepancies might be due either to the lack of consensus regarding the definition of cardiotoxicity (54) or to patient eligibility criteria, with some studies excluding patients with LVEF <55%, patients aged over 65 years, patients with cardiac risk factors, or those who had undergone mediastinal radiotherapy or hormone therapy. Thus, the question of whether cardiotoxicity is underestimated or not likely depends on the criterion chosen. It should be noted that in our study, the average decline in LVEF was 12 points (with criterion 1). Therefore, an absolute decrease of 10% in LVEF from baseline makes it possible to identify patients with an early decline in LVEF compared to other criteria. Although the evaluation of LVEF remains the reference for the measurement of cardiac dysfunction, the European Society of Cardiology (42) has recommended, since 2016, the assessment of GLS to identify subclinical lesions (55). Nevertheless, to date, as for LVEF, a consensual definition of GLS is still lacking, making it difficult to compare results between studies.

Currently, there is a paucity of data regarding the effects of exercise on the incidence of cardiotoxicity. Recent research reported the possible cardioprotective effects of exercise and its potential role in maintaining LVEF and GLS in BC patients undergoing chemotherapy or anti-HER2 antibodies (26, 51, 56). In our study, at inclusion, mean LVEF was 61.6% (SD: 6.9) in the TG and 59.6% (SD: 6.7) in the CG patients. These latter had completed surgery, chemotherapy and radiotherapy and were approximately 6 months from the beginning of treatment with trastuzumab. Similar data were found in the PHARE study (57) in which baseline LVEF was 66%. However, in that study, LVEF gradually decreased by 3.6% to reach a nadir at 12 months, in the absence of a physical exercise program (57). Thus, we can confirm that physical exercise, such as the program proposed in our study, may have tempered the decline in LVEF at T6. Besides, as suggested by Fei et al. (58), the maintenance of LVEF during follow-up might result either from the young age of our patients, who did not present heart failure and in whom cardiovascular risk was low because patients had completed their treatment with taxanes and/or anthracyclines, known to worsen cardiotoxicity. Here, the drop in LVEF was more important in patients who presented cardiotoxicity at T3 (−12 points [IQR: −14.0, −11.0]) than in patients without cardiotoxicity (+1.0 point [IQR: −3.0, 5.0]). These observations have previously been reported by Sendur et al. who found significant LVEF loss and higher cardiac biomarkers in patients having developed TIC (59).

Recently, a non-randomized study, conducted in women with early-stage BC who received usual care with or without physical training, showed a significant reduction in LVEF, but no difference after training in peak VO_2_ (26). Conversely, a randomized controlled study by Hornsby et al. reported that supervised aerobic training did not change LVEF, cardiac output, stroke volume, or diastolic and systolic volumes, but increased peak VO_2_, maximal power and pulse oxygen (60). Our results agree with this latter study since we found higher peak VO_2_ and maximal power after training without modification of LVEF and GLS.

To the best of our knowledge, available data exclusively in women with HER2 positive BC are scarce. Haykowsky et al. reported left ventricular dilation and a reduction in LVEF in HER2 positive BC patients treated by adjuvant trastuzumab, despite aerobic exercise training during the first 4 months of trastuzumab therapy (61). Furthermore, they did not observe any improvement in maximal power, peak VO_2_, heart rate, or perceived effort (61). Conversely in our study, the supervised exercise program was intermittent, with personalized target intensities (vs. continuous between 60–90% peak VO_2_) and the duration of sessions was longer in the study by Haykowsky et al. (55 vs. 30–60 min). Moreover, our program began at a distance from chemotherapy, while theirs was concomitant with chemo-radiotherapy.

More recently, Hojan et al. (33) did not observe any significant changes in LVEF or 6-minute walk distance after a 9-week exercise program. Although the intervention period took place at the same time as in our study (3 to 6 months after the beginning of trastuzumab), the training program was more substantial than ours, because it included supervised aerobic and weight training activities (90 min 5-day/week). However, our intervention was longer (12 vs. 9 weeks) and personalized with intensities determined by an exercise test (vs 80% according to the HRmax = 220–age), making it possible to improve cardiorespiratory capacities (such as peak VO_2_) and maintain cardiac parameters (LVEF and GLS).

Studies about left ventricular remodeling, cardiac events during trastuzumab therapy and clinical implications of fluctuations in LVEF are limited (11, 37). In patients with cardiac dysfunction, a slight decrease of LVEF may impact the physical ability to perform prolonged physical exercise, therefore impairing quality of life. More recently, studies conducted in patients with solid malignancies have shown that cardiorespiratory fitness, in particular peak VO_2_, is a predictor of anthracycline- and trastuzumab-induced left ventricular dysfunction (62–64). A slight decrease of LVEF following exposure to trastuzumab might alter the cardiorespiratory fitness of patients when intense physical activity is required. However, our study did find any correlation between changes in LVEF and peak VO_2_ or maximal power. These observations could be explained by the fact that the median values of LVEF remained stable over time, despite heterogeneous individual trajectories (see Figures 3, 4). Furthermore, while peak VO_2_ and maximal power were improved and followed the same trajectories in all the patients who participated in the exercise training, we did not observe variations in LVEF.

Moreover, peak VO_2_ and maximal power were comparable in patients with and in those without cardiac toxicity (TOX; *n* = 10, NoTOX; *n* = 65) while relative LVEF was significantly different at T3 between groups. We suggest that compensatory mechanisms, including preservation of absolute stroke volume, chronotropic competence, increased oxygen extraction or augmented pulmonary lymphatic flow (65) may have preserved exercise tolerance despite left ventricular dysfunction or remodeling. These mechanisms could partly explain the absence of difference in peak VO_2_ and maximal power between TOX and NoTOX groups and why LVEF changes did not influence cardiorespiratory fitness.

This study has some limitations that deserve to be underlined. First, diabetic patients or patients with an asymptomatic coronary pathology have not been identified in the study population, while these pathologies represent cardiotoxicity risk factors, like the side of radiotherapy that can affect the risk of cardiotoxicity development. In addition, it would have been interesting to study the markers in the measurement of cardiotoxicity such as those used in the ONCORE study (66). The use of a multimodal strategy that integrates several biomarkers with cardiac imaging could bring more information to detect early subclinical cardiotoxicity. Moreover, the number of subjects to meet the primary endpoint was not reached, and the overall population was likely too small to highlight significant effects in subgroup analyses. In addition, it would have been interesting to monitor the level of physical activity by actimetry during the first period (T0 to T3) in the CG and during the second period (T3 to T6) in both groups, to verify whether the total level of physical activity would have an effect on cardiotoxicity and physiological responses to exercise.

To conclude, a 12-week supervised exercise regimen was safe and improved the cardiopulmonary fitness in HER2-positive breast cancer patients treated with adjuvant trastuzumab therapy. The study is under powered to come to any conclusion regarding the effect on cardiotoxicity. Nevertheless, the lower limit of the binomial confidence interval in TG was higher than the null hypothesis rate (75%) confirming that this estimation was clearly higher than 75%. However, the results deserve further evaluations considering the use more important of targeted therapies, such as with trastuzumab emtansine in HER2-positive patients. Moreover, this study showed that exercise training enabled cardiopulmonary fitness, notably peak VO_2_, to be rapidly improved. However, the changes in cardiorespiratory capacities were not correlated with changes in LVEF, particularly in patients with cardiotoxicity. This indicates that resting measurements of cardiac parameters (LVEF and GLS) are not sensitive to change and are not correlated with functional disabilities induced by chemotherapy treatments. Finally, exercise training was well tolerated without side-effects. It may therefore provide additional benefits on top of the usual cancer treatment and prevent exacerbation of cardiac toxicities that occur as a result of trastuzumab treatment.

## Data availability statement

The raw data supporting the conclusions of this article will be made available by the authors, without undue reservation.

## Ethics statement

The studies involving human participants were reviewed and approved by Ethics Committee (Comité de Protection des Personnes Est-II), Besançon, France under the number P/2014/241 and by the National Health Products Safety Agency (N° ID RCB 2014-A01911-46). The patients/participants provided their written informed consent to participate in this study.

## Author contributions

Conception and design: QJ, NM, DV, and FM. Administrative support: QJ, NM, MB, PR, BD, DV, XP, and FM. Inclusion of patients: NM, EC, LM, M-JP, FB, LC, ED, GM, and XP. Collection and assembly of data: QJ, NM, PR, BD, MC, and FM. Data analysis and interpretation: QJ, NM, AF, DV, and FM. Manuscript writing: QJ, AF, DV, and FM. All authors contributed to the article and approved the submitted version.

## Funding

This study was supported by the Ligue Contre le Cancer association (CCIR-GE; N°8FI11826PYRO) through a 2014 Research Grant to FM.

## Conflict of interest

The authors declare that the research was conducted in the absence of any commercial or financial relationships that could be construed as a potential conflict of interest.

## Publisher's note

All claims expressed in this article are solely those of the authors and do not necessarily represent those of their affiliated organizations, or those of the publisher, the editors and the reviewers. Any product that may be evaluated in this article, or claim that may be made by its manufacturer, is not guaranteed or endorsed by the publisher.

## Abbreviations

ALAT: alanine aminotransferase
ASAT: aspartate transaminase
BC: Breast Cancer
BMI: body mass index
CG: Control group
ER: Estrogen Receptor
GLS: left ventricular global longitudinal strain
HER2: human epidermal growth factor receptor 2
HR: Heart rate
LV: left ventricular
LVEF: left ventricular ejection fraction
Peak VO_2_: Peak oxygen uptake
PR: Progesterone Receptor
SaO_2_: oxygen arterial saturation
TG: Training group
TIC: trastuzumab-induced cardiotoxicity
VO_2_/HR: Oxygen pulse
WHO: world health organization.
